# Interferon Gamma and Tumor Necrosis Factor Alpha Are Inflammatory Biomarkers for Major Adverse Cardiovascular Events in Patients with Peripheral Artery Disease

**DOI:** 10.3390/biomedicines13071586

**Published:** 2025-06-29

**Authors:** Ben Li, Eva Lindner, Raghad Abuhalimeh, Farah Shaikh, Houssam Younes, Batool Abuhalimeh, Abdelrahman Zamzam, Rawand Abdin, Mohammad Qadura

**Affiliations:** 1 Division of Vascular Surgery, St. Michael’s Hospital, Unity Health Toronto, University of Toronto, Toronto, ON M5B 1W8, Canada; benx.li@mail.utoronto.ca (B.L.);; 2 Department of Surgery, University of Toronto, Toronto, ON M5S 1A1, Canada; 3 Temerty Centre for Artificial Intelligence Research and Education in Medicine (T-CAIREM), University of Toronto, Toronto, ON M5S 1A1, Canada; 4 Institute of Medical Science, University of Toronto, Toronto, ON M5S 1A1, Canada; 5 Heart, Vascular, & Thoracic Institute, Cleveland Clinic Abu Dhabi, Abu Dhabi 112412, United Arab Emirates; 6 College of Medicine, University of Jordan, Amman 11942, Jordan; 7 Department of Medicine, McMaster University, Hamilton, ON L8S 4L8, Canada; 8 Li Ka Shing Knowledge Institute, St. Michael’s Hospital, Unity Health Toronto, University of Toronto, Toronto, ON M5B 1W8, Canada

**Keywords:** inflammatory proteins, tumor necrosis factor alpha, interferon gamma, major adverse cardiovascular events, prognosis, peripheral artery disease

## Abstract

**Background/Objectives:** Major adverse cardiovascular events (MACE)—including heart attacks and strokes—are the leading cause of death in patients with peripheral artery disease (PAD), yet biomarker research for MACE prediction in PAD patients remains limited. Inflammatory proteins play a key role in the progression of atherosclerosis and may serve as useful prognostic indicators for systemic cardiovascular risk in PAD. The objective of this study was to evaluate a broad panel of circulating inflammatory proteins to identify those independently associated with 2-year MACE in patients with PAD. **Methods:** We conducted a prospective cohort study involving 465 patients with PAD. Plasma concentrations of 15 inflammatory proteins were measured at baseline using validated immunoassays. Patients were followed over a two-year period for the development of MACE, defined as a composite endpoint of myocardial infarction, stroke, or mortality. Protein levels were compared between patients with and without MACE using the Mann–Whitney U test. Cox proportional hazards regression was used to determine the independent association of each protein with MACE after adjusting for baseline demographic and clinical variables, including existing coronary and cerebrovascular disease. To validate the findings, a random forest machine learning model was developed to assess the relative importance of each protein for predicting 2-year MACE. **Results:** The mean age of the cohort was 71 years (SD 10), and 145 participants (31.1%) were female. Over the two-year follow-up, 84 patients (18.1%) experienced MACE. Six proteins were significantly elevated in PAD patients who developed MACE: interferon gamma (IFN-γ; 42.55 [SD 15.11] vs. 33.85 [SD 12.46] pg/mL, *p* < 0.001), tumor necrosis factor alpha (TNF-α; 9.00 [SD 5.00] vs. 4.65 [SD 4.29] pg/mL, *p* < 0.001), chemokine (C-X-C motif) ligand 9 (CXCL9; 75.99 [SD 65.14] vs. 5.38 [SD 64.18] pg/mL, *p* = 0.002), macrophage inflammatory protein-1 beta (MIP-1β; 20.88 [SD 18.10] vs. 15.67 [SD 16.93] pg/mL, *p* = 0.009), MIP-1δ (25.29 [SD 4.22] vs. 17.98 [SD 4.01] pg/mL, *p* = 0.026), and interleukin-6 (IL-6; 12.50 [SD 40.00] vs. 6.72 [SD 38.98] pg/mL, *p* = 0.035). After adjusting for all baseline covariates, only two proteins—TNF-α (adjusted HR 1.66, 95% CI 1.28–2.33, *p* = 0.001) and IFN-γ (adjusted HR 1.25, 95% CI 1.12–2.29, *p* = 0.033)—remained significantly and independently associated with 2-year MACE. These findings were corroborated by the random forest model, where TNF-α and IFN-γ received the highest importance scores for predicting 2-year MACE: (TNF-α: 0.15 [95% CI 0.13–0.18], *p* = 0.002; IFN-γ: 0.19 [95% CI 0.17–0.21], *p* = 0.001). **Conclusions:** From a panel of 15 proteins, TNF-α and IFN-γ emerged as inflammatory biomarkers associated with 2-year MACE in PAD patients. Their measurement may aid in cardiovascular risk stratification, helping to identify high-risk individuals who could benefit from early multidisciplinary referrals to cardiology, neurology, and/or vascular medicine specialists to provide intensified medical therapy. Incorporating these biomarkers into PAD management may improve systemic cardiovascular outcomes through more personalized and targeted treatment approaches.

## 1. Introduction

Peripheral artery disease (PAD) is a manifestation of atherosclerosis affecting the arteries of the lower limbs and impacts over 200 million people worldwide [1]. While PAD is often recognized for its association with major adverse limb events (MALE), including limb loss, the leading cause of death among PAD patients is major adverse cardiovascular events (MACE), such as stroke and myocardial infarction (MI) [2]. This is due to the frequent coexistence of PAD with coronary artery disease (CAD) and cerebrovascular disease (CVD), conditions that share overlapping pathophysiological mechanisms [3]. These comorbidities are largely driven by common risk factors such as older age, diabetes, hypertension, hyperlipidemia, and smoking [4]. As a result, PAD often serves as a clinical marker for widespread vascular disease and systemic atherosclerosis [4]. Identifying which PAD patients are at greater risk for future MACE is critical to optimizing care, guiding specialist referrals, and implementing intensive risk-reduction therapies [5]. To date, however, clinical tools for stratifying cardiovascular risk in patients with PAD remain limited. One promising avenue involves the identification of circulating biomarkers that reflect the underlying pathobiology of systemic atherosclerosis [6,7,8,9]. Our group has previously identified inflammatory mediators associated with MALE in PAD patients [10]. However, less attention has been given to biomarkers that can predict MACE in this population. Bridging this gap may offer a valuable way to personalize cardiovascular risk assessment and improve systemic health outcomes in patients with PAD.

Emerging evidence underscores the pivotal role of the immune system in the pathogenesis of cardiovascular diseases through its involvement in systemic inflammation, atherosclerosis, and thrombosis [11]. In this context, circulating inflammatory proteins are of particular interest as potential biomarkers for MACE in patients with PAD, given their contributions to the molecular pathways driving systemic atherosclerosis [11]. Among these, interferon gamma (IFN-γ) and tumor necrosis factor alpha (TNF-α) have been shown to influence the initiation and progression of vascular inflammation and plaque instability, key mechanisms in the development of cardiovascular events [12,13]. Importantly, inflammation is linked to oxidative stress, a key driver of cardiovascular disease development and progression through its contribution to atherosclerosis, endothelial dysfunction, and thrombosis [14]. More broadly, over ten inflammatory proteins have been implicated in the pathophysiology of PAD, CAD, and CVD, suggesting a common inflammatory basis for these atherosclerotic conditions [15,16,17,18,19,20].

In this study, we selected 15 inflammatory proteins for analysis based on their strong associations with cardiovascular pathology and their biological plausibility as predictors of adverse outcomes in PAD [15,16,17,18,19,20]. While many of these proteins have been studied in relation to cardiovascular disease, few investigations have focused specifically on their prognostic utility for predicting MACE in PAD patients [15,16,17,18,19,20]. Previously, we demonstrated the diagnostic and prognostic value of inflammatory proteins in terms of identifying PAD and predicting MALE [10,21]. However, their role in predicting MACE in PAD patients remains poorly understood. The specific research gap is that there is a lack of investigation into inflammatory biomarkers for prognostication of MACE in PAD patients. The objective of this study was to assess the prognostic potential of a large panel of inflammatory biomarkers for 2-year MACE in a well-characterized PAD cohort. By identifying high-risk individuals through biomarker profiling, our goal is to support earlier intervention and more intensive risk management to prevent adverse cardiovascular events in the PAD population.

## 2. Materials and Methods

### 2.1. Ethics

Ethical approval was granted by the Unity Health Toronto Research Ethics Board on 8 February 2017 (REB #16-375). Prior to participation, all individuals provided informed consent. The study procedures adhered to the ethical principles established in the Declaration of Helsinki [22].

### 2.2. Design

This prognostic analysis was conducted and reported in accordance with the TRIPOD + AI guidelines [23].

### 2.3. Patient Recruitment

This study prospectively recruited consecutive patients with PAD who received care at the ambulatory vascular clinics of St. Michael’s Hospital between January 2018 and August 2019. The diagnosis of PAD was established based on a Toe-Brachial Index (TBI) of less than 0.7 or an Ankle-Brachial Index (ABI) of less than 0.9, in conjunction with absent or diminished pedal pulses [24]. All patients were classified as Fontaine stage IIA (mild claudication) or IIB (moderate–severe claudication), or Rutherford category 1 (mild claudication), 2 (moderate claudication), or 3 (severe claudication) [25]. Participants with Fontaine stage III (ischemic rest pain) or IV (ulceration or gangrene), or Rutherford category 4 (ischemic rest pain), 5 (minor tissue loss) or 6 (ulceration or gangrene) were excluded, as these patients would require vascular intervention at baseline [25]. These inclusion and exclusion criteria were intended to create a cohort of early-stage PAD patients with claudication, but without chronic limb-threatening ischemia, who would likely benefit from early and aggressive cardiovascular risk reduction strategies. Patients were also excluded if they had elevated troponin levels, acute limb ischemia, or acute coronary syndrome within the preceding three months. Additionally, patients with acute/chronic inflammatory disorders or autoimmune disorders were excluded.

### 2.4. Baseline Characteristics

In this study, we documented a range of baseline characteristics, including demographic factors (age and sex), established cardiovascular risk factors (hypertension, dyslipidemia, diabetes, chronic kidney disease [CKD] and smoking status), and a history of cardiovascular disease (CAD, congestive heart failure (CHF), and previous stroke). Hypertension was classified as either a systolic blood pressure ≥130 mmHg, a diastolic pressure ≥80 mmHg, or ongoing treatment with antihypertensive medications [26,27,28,29]. Dyslipidemia was defined by total cholesterol levels exceeding 5.2 mmol/L, triglyceride levels above 1.7 mmol/L, or current use of lipid-lowering drugs [26,27,28,29]. Diabetes (type 2) was identified by a hemoglobin A1c value of ≥6.5% or active treatment with antidiabetic agents [26,27,28,29]. CKD was defined as a glomerular filtration rate <60 mL/min/1.73 m^2^ or an albumin–creatinine ratio >30 mg/g for more than 3 months based on guidelines from the Kidney Disease Improving Global Outcomes (KDIGO) Work Group [30]. CHF was defined as a clinical syndrome with symptoms and/or signs caused by a structural and/or functional cardiac abnormality and corroborated by elevated natriuretic peptide levels and/or objective evidence of pulmonary or systemic congestion [26,27,28,29]. CAD was defined as the presence of atherosclerotic plaque in the coronary arteries [26,27,28,29]. Previous stroke was defined as a prior infarction of the brain, spinal cord, or retinal tissue due to ischemia, confirmed through either (a) objective evidence such as pathology or imaging identifying focal ischemic injury in a vascular territory, or (b) clinical symptoms of focal neurological dysfunction lasting ≥24 h or resulting in death, with other causes excluded [26,27,28,29]. Definitions of these risk factors were adapted from guidelines issued by the American College of Cardiology, American Heart Association, American Stroke Association, World Heart Federation, European Society of Cardiology, and KDIGO [26,27,28,29,30]. The use of cardiovascular risk reduction medications at recruitment, including statins, acetylsalicylic acid (ASA), and angiotensin-converting enzyme inhibitor (ACE-I) or angiotensin II receptor blocker (ARB), were also recorded. These medications were included because they can significantly affect inflammation, oxidative stress, and atherosclerotic plaque development [31,32].

### 2.5. Quantification of Plasma Protein Concentrations

Venous blood samples were collected from all participants, and plasma levels of 15 inflammatory markers were quantified in duplicate using a validated multiplex immunoassay (LUMINEX platform, Bio-Techne, Minneapolis, MN, USA) [33]. The selected biomarkers were chosen based on their known roles in inflammatory signaling pathways implicated in systemic atherosclerosis and their established relevance in cardiovascular conditions [15,16,17,18,19,20]. These proteins included IFN-γ, TNF-α, macrophage inflammatory protein-1 delta (MIP-1δ), chemokine (C-C motif) ligand 1 (CCL1), cluster of differentiation 163 (CD163), interleukin-6 (IL-6), resistin, chemokine (C-X-C motif) ligand 9 (CXCL9), monocyte chemoattractant protein-1 (MCP-1), eotaxin, progranulin, thymus and activation-regulated chemokine (TARC), CXCL16, MIP-1a, and MIP-1β. By assessing this diverse panel, the study aimed to uncover novel inflammatory biomarkers relevant to MACE risk in PAD patients. Prior to analyzing the samples, instrument calibration and performance validation were conducted using Fluidics Verification and Calibration bead kits (Luminex Corp, Austin, TX, USA) [34] on the MagPix analyzer (Luminex Corp, Austin, TX, USA) [35]. To ensure consistency, all assays were performed on a single day, minimizing variability. The intra- and inter-assay coefficients of variation were maintained below 10%. A minimum of 50 beads per analyte were acquired and evaluated using Luminex xPonent software version 4.3 [36].

### 2.6. Follow-Up and Outcomes

**Figure 1:**
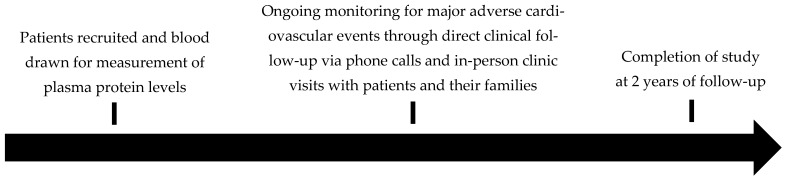
Research scheme for patient recruitment, follow-up, and assessment of major adverse cardiovascular events.

Participants attended routine follow-up appointments at 1 and 2 years after the initial assessment, with additional visits scheduled as necessary in response to clinical changes. The primary outcome evaluated was the occurrence of MACE over a two-year period. MACE was defined as a composite endpoint comprising stroke, MI, or mortality, determined through direct clinical evaluations and patient follow-up. MI was diagnosed based on a rise and/or fall in cardiac troponin levels, with at least one value exceeding the 99th percentile upper reference limit, accompanied by at least one of the following criteria: (a) symptoms consistent with myocardial ischemia, (b) new ischemic changes on an electrocardiogram, (c) development of pathological Q waves, (d) imaging evidence of new loss of viable myocardium or regional wall motion abnormality corresponding with an ischemic cause, or (e) angiographic or postmortem confirmation of a coronary thrombus [28]. This definition aligns with the joint guidelines of the World Heart Federation, American College of Cardiology, European Society of Cardiology, and American Heart Association [28]. Stroke was defined as infarction of the brain, spinal cord, or retinal tissue due to ischemia, confirmed through either: (a) objective evidence such as pathology or imaging identifying focal ischemic injury in a vascular territory, or (b) clinical symptoms of focal neurological dysfunction lasting ≥24 h or resulting in death, with other causes excluded [29]. This definition is consistent with recommendations from the American Stroke Association and American Heart Association [29]. All deaths recorded were classified as all-cause mortality. Although both 1-year and 2-year MACE were captured, this study focused on 2-year MACE as it provides longer-term follow-up, thus highlighting the more sustained prognostic value of the inflammatory proteins. Figure 1 illustrates the research scheme for patient recruitment, follow-up, and assessment of 2-year MACE over the study period.

### 2.7. Statistical Analysis

Descriptive statistics were used to summarize the baseline characteristics and clinical event rates in the study population, reported as means with standard deviations for continuous variables, and counts with corresponding percentages for categorical variables. Differences in plasma protein levels between PAD patients who experienced MACE within 2 years and those who did not were evaluated using the non-parametric Mann–Whitney U test. Proteins that exhibited significant expression differences between these groups were further analyzed to assess their prognostic value.

To investigate the relationship between protein concentrations and the risk of 2-year MACE, Cox proportional hazards models were employed, adjusting for potential confounders including age, sex, hypertension, dyslipidemia, diabetes, CKD, smoking status (past or current), CHF, CAD, prior stroke, and use of cardiovascular risk reduction medications.

To complement the Cox regression and account for potential nonlinearities and interactions, a random forest machine learning approach was also applied [37]. This model incorporated the same clinical covariates and protein measurements to predict the occurrence of 2-year MACE [37]. Random forest modeling is well-suited for identifying complex, nonlinear associations and does not rely on the proportional hazards assumption, making it a valuable tool for validating statistical findings [37]. The random forest consisted of 100 decision trees and employed bootstrapping techniques to enhance model stability [37].

A two-sided *p*-value of less than 0.05 was considered statistically significant. All statistical analyses were performed using SPSS version 23 (SPSS Inc., Chicago, IL, USA) [38].

## 3. Results

### 3.1. Patient Characteristics

The study cohort consisted of 465 individuals diagnosed with PAD. The average age of participants was 71 years (SD 10), and women made up 31.1% of the sample (*n* = 145). Hypertension was present in 84.6% of patients, while 82.3% had dyslipidemia. Nearly half of the cohort (47.2%) had diabetes or CKD (46.5%), 57.9% reported a history of smoking, and 23.6% were current smokers. Additionally, 4.7% of participants had a diagnosis of CHF, 39.0% had established CAD, and 19.7% had a prior history of stroke. In terms of risk reduction medications, 75.3% of patients received ASA, 69.2% received a statin, and 62.4% received an ACE-I/ARB. A detailed breakdown of baseline characteristics is provided in Table 1. The clinically meaningful finding was the high burden of cardiovascular risk factors in the PAD population.

### 3.2. Outcomes

Over the course of the 2-year follow-up, MACE was observed in 84 patients, accounting for 18.1% of the study population. Among these events, MI was the most frequent, occurring in 70 individuals (15.0%). Stroke was documented in 22 patients (4.7%), while 5 patients (1.2%) died from any cause during the follow-up period. At 1 year of follow-up, 52 (11.2%) patients developed MACE, including the following components: 45 (9.7%) MIs, 13 (2.8%) strokes, and 3 (0.6%) deaths. These outcome details are summarized in Table 2. The clinically meaningful finding was the relatively high incidence of 1-year and 2-year MACE in the PAD population.

### 3.3. Plasma Concentrations of Inflammatory Proteins

Out of the 15 inflammatory proteins examined, 6 were found to be significantly elevated in patients with PAD who developed MACE within 2 years, compared to those who did not experience these events. These proteins included IFN-γ, TNF-α, CXCL9, MIP-1β, MIP-1δ, and interleukin-6 (IL-6). Detailed results are presented in Table 3.

### 3.4. Associations Between Inflammatory Proteins and 2-Year MACE in Patients with PAD

After adjusting for demographic and clinical risk factors, elevated plasma levels of TNF-α and IFN-γ emerged as independent predictors of 2-year MACE among patients with PAD. Specifically, TNF-α was associated with a higher risk of MACE, with an adjusted hazard ratio (HR) of 1.66 (95% CI: 1.28–2.33; *p* = 0.001), while IFN-γ showed a significant association as well, with an adjusted HR of 1.25 (95% CI: 1.12–2.29; *p* = 0.033) (Table 4). These findings were further supported by random forest modeling, which identified IFN-γ and TNF-α as the most informative biomarkers, yielding importance scores of 0.19 (95% CI: 0.17–0.21; *p* = 0.001) and 0.15 (95% CI: 0.13–0.18; *p* = 0.002), respectively, even after accounting for all baseline variables (Table 5). The clinically and statistically meaningful finding was that IFN-γ and TNF-α were important and independent predictors of 2-year MACE in patients with PAD. Patients with higher plasma levels of these inflammatory proteins were more likely to have 2-year MACE during the follow-up period.

## 4. Discussion

### 4.1. Summary of Study Findings

This study highlights IFN-γ and TNF-α as inflammatory biomarkers that are independently associated with the risk of MACE within 2 years among individuals diagnosed with PAD, suggesting their potential utility in prognostic assessment. Our analysis yielded several important insights. First, among the 15 plasma inflammatory proteins evaluated, 6 (IFN-γ, TNF-α, CXCL9, MIP-1β, MIP-1δ, and IL-6) were found at significantly higher levels in MACE vs. non-MACE patients. Second, of these six markers, only IFN-γ and TNF-α retained a statistically significant association with 2-year MACE after adjustment for demographic variables and traditional cardiovascular risk factors, including existing CAD and CVD. Third, the prognostic relevance of IFN-γ and TNF-α was confirmed using a machine learning approach—specifically, a random forest classifier—which reaffirmed their importance in predicting adverse cardiovascular outcomes in PAD patients. Collectively, these findings suggest that IFN-γ and TNF-α may offer valuable insights into cardiovascular risk stratification in the PAD population, potentially enabling more personalized and proactive clinical management.

### 4.2. Comparison to Existing Literature

Elyasi et al. (2020) conducted a comprehensive review of IFN-γ’s involvement in cardiovascular pathology, emphasizing its integral role in every phase of atherosclerotic progression—from the recruitment of immune cells to the accumulation of modified low-density lipoprotein (LDL) and eventual plaque formation and stabilization [12]. One critical mechanism by which IFN-γ contributes to atherogenesis is through its disruption of cholesterol transport systems, favoring the transformation of macrophages into foam cells, a hallmark of early plaque development [39,40]. As the principal cytokine secreted by type 1 helper T cells (Th1), IFN-γ has been consistently detected in atherosclerotic coronary arteries [41,42]. Notably, Th1 cells represent the dominant T-cell subtype within atherosclerotic lesions [43,44]. Extending this understanding to PAD, Zhao et al. (2024) identified a link between circulating IFN-γ levels and the onset of PAD [45], while Botti et al. (2012) reported elevated serum IFN-γ concentrations in individuals diagnosed with PAD [46]. Our findings build upon this prior work by illustrating that IFN-γ not only correlates with the presence of PAD but also predicts adverse cardiovascular outcomes in affected individuals, particularly MACE, within two years.

Rolski and colleagues (2020) detailed the pathophysiological role of TNF-α in cardiovascular conditions, highlighting its central involvement in a wide array of inflammatory processes contributing to vascular disease [47]. Similarly, Yuan et al. (2020) demonstrated that elevated circulating TNF-α levels were positively associated with CAD and ischemic stroke risk [13]. Pande and colleagues demonstrated an association between TNF-α inflammatory gene expression in peripheral blood monocytes with walking impairment in patients with PAD and intermittent claudication [48]. This suggests that higher circulating TNF-α levels may be associated with muscle ischemia during exertion in patients with PAD [48]. Ridker et al. further emphasized TNF-α’s significance by showing that persistently elevated levels are seen in post-MI patients who are at an increased risk for recurrent events [49]. This study highlights that there is persistent inflammatory instability characterized by increased TNF-α levels among patients with an increased risk of MACE [49]. In alignment with these findings, our study establishes a strong relationship between TNF-α concentrations and future cardiovascular complications in PAD patients, reinforcing its role in the broader spectrum of systemic atherosclerotic disease.

Prior work by our group demonstrated associations between inflammatory markers and limb-related complications in PAD [10]. By extending this to systemic cardiovascular events, our findings underscore the dual role of IFN-γ and TNF-α not only in PAD pathology but also in predicting broader cardiovascular outcomes. These results underscore the need for deeper investigation into the immunological mechanisms underpinning PAD, CAD, and CVD. A better understanding of these inflammatory pathways involving proteins like IFN-γ and TNF-α may ultimately inform the development of novel, targeted therapies aimed at reducing the burden of atherosclerotic disease across multiple vascular beds.

### 4.3. Explanation of Findings

Several underlying biological mechanisms likely explain the associations observed between elevated IFN-γ and TNF-α levels and increased 2-year MACE risk in patients with PAD. IFN-γ plays a central role in both innate and adaptive immunity and is the only member of the type II interferon family [50]. Compared to type I interferons, IFN-γ is markedly more potent, exerting immunomodulatory effects up to 10,000 times greater [51]. This cytokine consists of 143 amino acids, has a molecular weight of approximately 17 kDa, and is functionally active as a non-covalent homodimer [52]. The two monomers align in an antiparallel orientation—one’s N-terminus paired with the other’s C-terminus [52]. Structural analyses reveal that each IFN-γ monomer is composed of six α-helices, linked by flexible regions, and capped by a 21-residue C-terminal segment [53]. Cleavage of the C-terminus up to nine amino acid residues leads to an increase in IFN-y receptor binding, but further removal of the tail leads to inactivation of the entire cytokine [53]. Primarily secreted by activated macrophages and Th1 cells, IFN-γ orchestrates a wide spectrum of immune responses, including the modulation of more than 2300 genes involved in inflammation and cell-mediated immunity [12]. It exerts many of its effects through the Janus kinase-signal transducer and activator of transcription signaling cascade to induce oxidative stress, promote foam cell accumulation, stimulate smooth muscle cell proliferation and migration into the arterial intima, enhance platelet-derived growth factor expression, and destabilize plaque [12]. Release of IFN-y activates multiple cells of the innate and adaptive immune response, including natural killer cells, B cells, monocytes, macrophages, and vascular endothelial cells [54]. This launches a cascade of proinflammatory molecules that recruit monocytes to the endothelial wall where IFN-y increases the expression of adhesion molecules through the activation of endothelial cells [55,56]. Once monocytes are chemoattracted to the vessel wall, they can breach the activated endothelial cell monolayer, settle in the subintima, differentiate into macrophages, and consume oxidized LDL via scavenger receptors [57,58]. IFN-y therefore increases scavenger receptor expression, encouraging macrophage lipid overload and foam cell transformation [57,58]. Given that these mechanisms contribute significantly to the initiation and progression of atherosclerotic disease, they may explain why IFN-y was shown to be an important predictor of 2-year MACE in patients with PAD [12].

TNF-α is another proinflammatory cytokine with broad relevance in cardiovascular pathology. Encoded by the TNF-A gene on chromosome 6, human TNF-α shares a locus with major histocompatibility complex class II genes [59]. The TNF-A gene consists of 200 nucleotide promoters with binding sites for several transcription factors, resulting in a high plasticity of transcription and responsiveness to various types of stimuli [59]. Upon translation, TNF-α is synthesized as a 17 kDa type II transmembrane protein, possessing a single, uncleavable transmembrane segment, which anchors the protein in a cell membrane with the C-terminal end oriented toward the cytoplasm [60]. This membrane form can act locally or be cleaved by TNF-α cleaving enzyme and be released as soluble TNF-α to exert systemic effects [60]. In cardiomyocytes, TNF-α impairs calcium cycling, elevates oxidative stress, and disrupts mitochondrial function, which together promote contractile dysfunction, apoptosis, and maladaptive remodeling such as hypertrophy and necroptosis [61,62]. In the vascular endothelium, TNF-α engages its receptors, TNF receptors 1 and 2, initiating signaling pathways that elevate reactive oxygen species production and upregulate expression of adhesion molecules, thereby enhancing endothelial permeability and facilitating LDL transcytosis [63,64]. These vascular changes compromise endothelial barrier integrity, favor lipid infiltration, and foster immune cell recruitment [63,64]. These pro-atherogenic effects reinforce the role of TNF-α as a driver of cardiovascular pathology and support its predictive value for MACE in patients with PAD [63,64]. Collectively, these molecular mechanisms highlight the central roles of IFN-γ and TNF-α in systemic inflammation and atherogenesis, providing a biological rationale for their identification as prognostic biomarkers for systemic cardiovascular risk in patients with PAD.

### 4.4. Implications

This study offers important clinical insights that can enhance decision-making for patients diagnosed with PAD. Measuring circulating levels of IFN-γ and TNF-α could serve as a practical tool for predicting MACE risk, especially within primary care settings [65]. Family physicians, who often serve as the first point of contact in the healthcare system, can incorporate these biomarkers into routine evaluations to stratify cardiovascular risk in PAD patients [65]. For individuals identified as having an elevated biomarker profile—and therefore a higher predicted risk of MACE—timely referral to appropriate specialists such as neurologists, cardiologists, or vascular medicine experts may be warranted to initiate more comprehensive diagnostic workups and therapeutic planning [66]. Conversely, patients categorized as low-risk could continue to receive longitudinal management in the primary care setting, focusing on conventional secondary prevention strategies [67]. These include pharmacologic interventions such as aspirin and statins, along with lifestyle interventions aimed at optimizing modifiable risk factors, including smoking cessation, dietary improvement, and regular physical activity [67]. For those referred to specialists, our findings provide a basis for more personalized treatment strategies based on anticipated cardiovascular risk. For instance, recent evidence supports the addition of low-dose rivaroxaban to aspirin to reduce cardiovascular events in patients with stable CAD or PAD [68]. In high-risk individuals, advanced imaging techniques—such as coronary or cerebrovascular angiography—may be warranted to detect subclinical but hemodynamically significant lesions that could benefit from early intervention [69]. Broadly, integrating IFN-γ and TNF-α testing into routine care could enable a more nuanced approach to PAD management, guiding resource allocation, optimizing referrals, and informing therapeutic intensity, ultimately improving patient outcomes [70].

### 4.5. Limitations

This study is not without limitations, several of which should be acknowledged to contextualize the findings. First, the research was carried out at a single academic institution, which limits the generalizability of the results. Validation in diverse, multi-center cohorts is essential to confirm the applicability of these findings across varied clinical populations and healthcare settings. Second, the follow-up duration was limited to two years, which may not fully capture the long-term prognostic implications of IFN-γ and TNF-α, particularly in the context of chronic, progressive diseases such as PAD, CAD, and CVD. Longitudinal studies with extended follow-up periods are needed to assess the long-term prognostic value of these biomarkers. Third, the 15 inflammatory proteins were selected for analysis in this study because of their strong associations with cardiovascular pathology and their biological plausibility as predictors of adverse outcomes in PAD [15,16,17,18,19,20]. However, we acknowledge that other inflammatory proteins may be potential prognostic markers for systemic cardiovascular risk and were outside the scope of this study, such as IL-18 [71]. Such proteins were not included in this study to reduce the risk of finding spurious associations in a large number of statistical comparisons [71]. It would be prudent to investigate these proteins in future work. Furthermore, blood was only drawn at the beginning of the study for baseline quantification of circulating protein levels and not re-measured throughout the study. This was because our goal was to assess the prognostic value of the protein biomarkers at the beginning of the 2-year follow-up period to ensure sufficient time for adequate and effective risk reduction therapies. However, future studies assessing the changes in plasma protein concentrations over time, particularly given the variability and volatility of oxidative stress and local inflammation, would allow for further characterization of the longitudinal prognostic value of the biomarkers. Additionally, high-sensitivity C-reactive protein, lipid profiles, and white blood cell counts were not measured in this study [72,73]. Including these blood tests in future inflammatory biomarker-based studies may yield further mechanistic insights [72,73]. Moreover, the relationship between PAD severity and plasma levels of the inflammatory proteins was not captured and would be an important area of future investigation. In terms of how drugs evaluated in this study might affect the clinical outcomes and the plasma concentrations of the inflammatory proteins over time, we did not have sufficient data to adequately address these questions, which would be important for future research. Fourth, it is important to note that measurement of plasma IFN-γ and TNF-α is largely limited to research settings and has yet to be adopted in routine clinical workflows. Bridging this gap will require focused translational studies and implementation science to assess the practicality, cost-effectiveness, and clinical impact of incorporating these biomarkers into standard care pathways for the management of patients with PAD.

## 5. Conclusions

This study identified IFN-γ and TNF-α as circulating inflammatory proteins independently correlated with 2-year MACE in PAD patients, demonstrating their potential role as prognostic biomarkers. The predictive significance of these biomarkers was further validated through a random forest machine learning model, underscoring their utility in cardiovascular risk stratification. These findings pave the way for improved personalized care in PAD by enabling early identification of high-risk individuals who may benefit from timely, multidisciplinary intervention for intensive cardiovascular risk reduction. This is of particular relevance given the high mortality burden in PAD populations due to MI and stroke. Additionally, the study emphasizes the importance of continued research into the biological pathways linking IFN-γ and TNF-α to the progression of systemic atherosclerosis. A deeper mechanistic understanding could drive the development of targeted therapies aimed at mitigating systemic cardiovascular risk in patients with PAD.

## Figures and Tables

**Table 1:** Baseline characteristics.

	Patients with PAD (*n* = 465)
Age, mean (SD)	71 (10)
Female sex	145 (31.1)
Hypertension	394 (84.6)
Dyslipidemia	383 (82.3)
Diabetes	220 (47.2)
Chronic kidney disease	216 (46.5)
Past smoking	269 (57.9)
Current smoking	110 (23.6)
Congestive heart failure	22 (4.7)
Coronary artery disease	181 (39.0)
Previous stroke	92 (19.7)
Acetylsalicylic acid	350 (75.3)
Statin	322 (69.2)
ACE-I/ARB	290 (62.4)

Values are reported as numbers (%) unless stated otherwise. Abbreviations: SD (standard deviation), PAD (peripheral artery disease), ARB (angiotensin II receptor blocker), and ACE-I (angiotensin-converting enzyme inhibitor).

**Table 2:** Major adverse cardiovascular events at 1 year and 2 years of follow-up.

Patients with PAD (*n* = 465)	1 Year of Follow-Up	2 Years of Follow-Up
Major adverse cardiovascular event	52 (11.2)	84 (18.1)
Myocardial infarction	45 (9.7)	70 (15.0)
Stroke	13 (2.8)	22 (4.7)
Death	3 (0.6)	5 (1.2)

Values are reported as numbers (%). Abbreviations: SD (standard deviation); PAD (peripheral artery disease).

**Table 3:** Plasma protein concentrations.

	No MACE (*n* = 381)	MACE (*n* = 84)	*p*-Value
**IFN-γ**	**33.85 (SD 12.46) pg/mL**	**42.55 (SD 15.11) pg/mL**	**<0.001**
**TNF-α**	**4.65 (SD 4.29) pg/mL**	**9.00 (SD 5.00) pg/mL**	**<0.001**
**CXCL9**	**5.38 (SD 64.18) pg/mL**	**75.99 (SD 65.14) pg/mL**	**0.002**
**MIP-1β**	**15.67 (SD 16.93) pg/mL**	**20.88 (SD 18.10) pg/mL**	**0.009**
**MIP-1δ**	**17.98 (SD 4.01) pg/mL**	**25.29 (SD 4.22) pg/mL**	**0.026**
**IL-6**	**6.72 (SD 38.98) pg/mL**	**12.50 (SD 40.00) pg/mL**	**0.035**
TARC	298.95 (SD 426.3) pg/mL	273.06 (SD 263.81) pg/mL	0.562
MIP-1a	2.85 (SD 2.21) pg/mL	2.96 (SD 2.11) pg/mL	0.640
MCP-1	652.15 (SD 1051.28) pg/mL	606.93 (SD 849.17) pg/mL	0.694
CCL1	2.84 (SD 3.05) pg/mL	2.74 (SD 1.4) pg/mL	0.763
CXCL16	12.21 (SD 11.51) pg/mL	12.03 (SD 10.71) pg/mL	0.897
Eotaxin	122.91 (SD 179.21) pg/mL	125.44 (SD 59.97) pg/mL	0.893
Resistin	4.27 (SD 5.35) pg/mL	4.33 (SD 4.88) pg/mL	0.912
CD163	119.75 (SD 170.21) pg/mL	121.24 (SD 163.75) pg/mL	0.942
Progranulin	17.23 (SD 29.31) pg/mL	171.13 (SD 19.90) pg/mL	0.970

Protein concentrations reported as mean (standard deviation) in pg/mL. Bolded rows are statistically significant (*p* < 0.05). Abbreviations: interferon gamma (IFN-γ), tumor necrosis factor alpha (TNF-α), macrophage inflammatory protein-1 delta (MIP-1δ), macrophage inflammatory protein-1 beta (MIP-1β), macrophage inflammatory protein-1 alpha (MIP-1a), chemokine (C-C motif) ligand 1 (CCL1), cluster of differentiation 163 (CD163), interleukin 6 (IL-6), chemokine (C-X-C motif) ligand 9 (CXCL9), chemokine (C-X-C motif) ligand 16 (CXCL16), monocyte chemoattractant protein-1 (MCP-1), thymus and activation-regulated chemokine (TARC), MACE (major adverse cardiovascular event), SD (standard deviation), pg/mL (picograms per milliliter).

**Table 4:** Associations between inflammatory proteins and 2-year major adverse cardiovascular events.

	Adjusted Hazard Ratio (95% CI)	*p*-Value
**TNF-α**	**1.66 (1.28–2.33)**	**0.001**
**IFN-γ**	**1.25 (1.12–2.29)**	**0.033**
CXCL9	1.01 (0.93–1.07)	0.367
MCP-1	1.13 (0.91–1.20)	0.408
MIP-1a	1.29 (0.64–1.38)	0.423
CCL1	1.19 (0.96–1.30)	0.674
MIP-1δ	1.02 (0.86–1.19)	0.680
MIP-1β	1.10 (0.83–1.19)	0.687
IL-6	1.18 (0.63–1.27)	0.718
Eotaxin	0.92 (0.76–1.37)	0.728
CXCL16	1.19 (0.72–1.37)	0.742
Progranulin	1.17 (0.83–1.30)	0.797
TARC	1.12 (0.95–1.26)	0.811
CD163	1.18 (0.96–1.30)	0.871
Resistin	0.94 (0.76–1.11)	0.885

Bolded rows are statistically significant (*p* < 0.05). Abbreviations: interferon gamma (IFN-γ), tumor necrosis factor alpha (TNF-α), macrophage inflammatory protein-1 delta (MIP-1δ), macrophage inflammatory protein-1 beta (MIP-1β), macrophage inflammatory protein-1 alpha (MIP-1a), chemokine (C-C motif) ligand 1 (CCL1), cluster of differentiation 163 (CD163), interleukin 6 (IL-6), chemokine (C-X-C motif) ligand 9 (CXCL9), chemokine (C-X-C motif) ligand 16 (CXCL16), monocyte chemoattractant protein-1 (MCP-1), thymus and activation-regulated chemokine (TARC), and CI (confidence interval).

**Table 5:** Importance scores for inflammatory proteins in random forest model for predicting 2-year major adverse cardiovascular events.

	Importance Score	95% CI	*p*-Value
**IFN-γ**	**0.19**	**(0.17–0.21)**	**0.001**
**TNF-α**	**0.15**	**(0.13–0.18)**	**0.002**
MIP-1δ	0.15	(0.13–0.17)	0.231
MIP-1β	0.15	(0.11–0.16)	0.235
CD163	0.14	(0.12–0.18)	0.244
IL-6	0.14	(0.10–0.19)	0.002
Resistin	0.13	(0.10–0.15)	0.002
CXCL9	0.13	(0.11–0.15)	0.270
MCP-1	0.12	(0.10–0.14)	0.306
Eotaxin	0.10	(0.08–0.12)	0.370
CCL1	0.09	(0.07–0.11)	0.391
TARC	0.08	(0.06–0.10)	0.447
Progranulin	0.08	(0.06–0.10)	0.468
CXCL16	0.06	(0.04–0.08)	0.537
MIP-1a	0.06	(0.04–0.08)	0.550

Bolded rows are statistically significant (*p* < 0.05). Abbreviations: interferon gamma (IFN-γ), tumor necrosis factor alpha (TNF-α), macrophage inflammatory protein-1 delta (MIP-1δ), macrophage inflammatory protein-1 beta (MIP-1β), macrophage inflammatory protein-1 alpha (MIP-1a), chemokine (C-C motif) ligand 1 (CCL1), cluster of differentiation 163 (CD163), interleukin 6 (IL-6), chemokine (C-X-C motif) ligand 9 (CXCL9), chemokine (C-X-C motif) ligand 16 (CXCL16), monocyte chemoattractant protein-1 (MCP-1), thymus and activation-regulated chemokine (TARC), and CI (confidence interval).

## Data Availability

The original contributions presented in the study are included in the article; further inquiries can be directed to the corresponding author.
